# 

**Published:** 1916-08-05

**Authors:** 


### St. Thomas's Hospital.

House Appointments.—The following have been selected as house officers from Tuesday, August 8, 1916 (subject to the approval of the treasurer): *Casualty Officers and Resident Anæsthetists*: E. H. V. Hensley, B.A. Cantab., M.R.C.S., L.R.C.P.; M. St. C. Hamilton, M.R.C.S., L.R.C.P.; N. A. Sprott, B.A., M.B., B.Ch. Oxon., M.R.C.S., L.R.C.P.; A. G. P. Wills, B.A. Cantab., M.R.C.S., L.R.C.P.; R. C. P. Whitcombe, B.A. Cantab., M.R.C.S., L.R.C.P. *Resident House Physicians*: A. Mavrogordato, M.A. Oxon., M.R.C.S., L.R.C.P.; O. H. Hyman; J. R. Harris. *Resident House Surgeons*: D. C. Bluett; W. Marriott; S. H. M. Johns, M.A. Cantab., M.R.C.S., L.R.C.P.; F. H. Vey. *House Surgeon to Block 8 and Resident Anæsthetist*: O. H. Hyman.

### Literary Intelligence.

A NEW BOOK ON GYNÆCOLOGY.

A NEW volume by Dr. T. W. Eden and Dr. C. Lockyer, obstetric physicians to Charing Cross Hospital, entitled "Gynæcology for Students and Practitioners," has just been published by Messrs. J. and A. Churchill, price 27s. net. The illustrations, which are numerous, have been drawn in most cases from originals in the Lockyer collection in the museum of Charing Cross Hospital Medical School.

"THE CHILD WELFARE ANNUAL."

DR. T. N. KELYNACK is to be congratulated on the acceptance by Her Majesty the Queen of a copy of the *Child Welfare Annual*, of which he is the editor and Messrs. John Bale, Sons and Danielsson, Ltd., the publishers. The acceptance was accompanied by the following appreciation: "The Queen has inspected the volume with much interest, and Her Majesty feels sure that a work of this important nature will prove of the greatest assistance in this critical period of our existence. The Queen congratulates all those who have co-operated in the preparation of the *Child Welfare Annual* upon the successful result of their efforts."

A NEW BOOK ON HERB-GROWING.

"COUNTRY LIFE," LTD., are publishing this month in their "Country Life" Library a new book by Miss A. B. Teetgen. It is entitled "Profitable Herb-growing and Collecting," and its price will be 3s. 6d.

### The Book Market.

LIST OF NEW BOOKS RECEIVED FROM THE FOLLOWING PUBLISHERS:—

Erskine Macdonald: "Songs of Protest." By Frederick Mitchell. 1s. net.

The National Clean Milk Society: "Campaign for Clean Milk." 1s. net.

The Prudential Press, Newark, N.J., U.S.A.: "The Mortality from Cancer throughout the World."

Smith, Elder and Co.: "More Minor Horrors." By A. E. Shipley, Sc.D. 1s. 6d. net.

Baillière, Tindall and Cox: "After-Treatment of Operations." By P. Lockhart Mummery, F.R.C.S. Fourth Edition. 5s. net.

Percy Lund, Humphries and Co., Ltd., Bradford: "Surgical Bandages and Dressings," with Diagrams. By Alice Scott. 3s. 6d. net.

W. Thacker and Co.: "Handbook for Wives and Mothers in India." By Mildred E. Staley, M.B., L.M. 5s. net.

### HOSPITAL AND INSTITUTIONAL RESULTS IN 1915.

**Royal Infirmary, Sunderland.**—The report contains 140 pages; 1,145 sick and wounded sailors and soldiers have been admitted since the outbreak of the war. Total receipts, £17,080, include £7,182 workpeople's contributions, £205 legacies, and £3,443 in-patients' payments. Total expenditure, £15,625.

**Samaritan Free Hospital.**—A neighbouring freehold house, with a large hall attached, has been bought, as the hall had been let as a social club, the noise from which seriously interfered with the health of the patients. Total income (including legacies £814), £5,992; total expenditure, £6,274.

**Seamen's Hospital Society, Greenwich.**—A smiling member of the Naval Division, who has lost one leg, lends his portrait to the cover of the report, and within we find a photograph of the scene when the King and Queen visited the hospital on September 21, 1915. The total income was £28,619 (including £1,944 legacies and £6,091 patients' payments); the total expenditure was £23,718.

**Warneford, Leamington, and South Warwickshire Hospital.**—There are forty-four members of the committee of management, not counting a number *ex-officio*; 360 soldiers were treated in the wards and 524 as out-patients. £13,500 has been received under the will of the late Mr. Edward Wright, and the total legacies for the year were £14,697. Ordinary income, £6,139; total expenditure, £6,684.

**Wolverhampton and Staffordshire General Hospital.**—In-patients decreased slightly and out-patients considerably. The cost per occupied bed rose from £64 0s. 11d. to £77 3s. 2d. The total income, £15,607, included £4,584 from the Hospital Saturday Fund; the expenditure was £15,327.

**Monmouthshire Asylum.**—At the close of the year there were 629 male and 631 female patients. The report of the Visiting Commissioner states that there had been no employment of mechanical restraint or seclusion since the last visit, and that the weekly maintenance rate for home patients was 9s. 4d. Epileptics amounted to 13.9 per cent. of the total in residence.

**St. Mary's Convalescent Home, Birchington-on-Sea.**—Up to the time of the issue of the report the home could congratulate itself on preservation from air raids, and we hope this immunity will continue. Two hundred and seventy patients were received, including seventy-three mothers and babies (five pairs of twins) and forty-five ladies (presumably not mothers), "making in all 388 inmates during the year." Total income £754, total expenditure, £800.

**Cumberland and Westmorland Lunatic Asylum.**—959 patients were in the asylum at the end of the year. The recovery rate was 35.6 per cent. in 1915. The institution is considerably overcrowded in consequence of the taking over of the Newcastle City Asylum for use as a military hospital. The dinner served during the Commissioners' visit "consisted of good helpings of boiled rice and rhubarb tart, with bread and cheese, and was much appreciated."

**County and City of Worcester Lunatic Asylum, Powick.**—The asylum, like many other asylums, has taken extra patients to enable another institution to be used as a military hospital; there were 1,327 in the wards in March last as compared with a normal 1,000. The report of the Commissioner of the Board of Control is of a satisfactory nature. Eighty-three patients were discharged in 1915. The average weekly cost of each patient is stated to be 9s. 5½d.

**Convalescent Home for Poor Children, St. Leonards-on-Sea.** — Seven hundred and fifty-two boys and girls were admitted during the year, many of them children of sailors and soldiers. Total income, including £367 from invested funds and a legacy of £25, £1,769; total expenditure, £2,072.

**National Sanatorium Association.**—The Association is "for the establishment and maintenance of sanatoria for workers suffering from tuberculosis," and the National Sanatorium at Benenden is the result of its organisation. The total income was £6,929, derived from the Post Office Association, King Edward's Hospital Fund, the Hospital Saturday Fund, Friendly Societies, and Insurance Committees. The profit on farm work amounted to £369. The total expenditure was £6,555.

**Winsley Sanatorium for Consumption.** — The sanatorium, situated near Bath, serves several western counties. Three hundred and ninety-two patients were received in 1915, of whom 324 were discharged "fit for some work" or improved. Total income, £8,244; total expenditure, £7,503.

### PASSING EVENTS AND TOPICS.

### Needlework Exhibition.

To assist Red Cross funds the *Daily Sketch* is organising a second Needlework Exhibition, when £1,000 will be given in prizes. A special class has been arranged for wounded soldiers.

### A Government Grant.

On condition that the institution is available under the Roxburghshire scheme for the treatment of tuberculous patients under the National Insurance Act, a Government grant of £180 has been provided towards the cost of the additions to the Anderson Sanatorium, Hawick.

### Glasgow's Gift of a Dental Ambulance.

The Lord Provost of Glasgow has collected £1,112 for the provision of a completely equipped dental ambulance, which will bear Glasgow's name and be in charge of a staff from the city, as a gift to the French Red Cross. The sum obtained will also cover the maintenance cost for some months.

### Party Politics Must Go.

Because the chairman of the Health Committee, Alderman Dr. Benson, was allowed to make a protest against the Government's reduction of the fee for the notification of infectious diseases from 2s. 6d. to 1s., the Conservative and Labour members of the Wigan Town Council walked out of the Council Chamber in a body.

### L.G.B. and a Tuberculosis Scheme.

The Local Government Board has come down rather heavily on the tuberculosis scheme prepared by the Forfar District Committee. It has intimated very plainly that the committee must agree to deal with Poor-Law cases, otherwise the Board will not approve the scheme. In the latter event no share of the tuberculosis maintenance grant will be payable to the committee.

### Offer to Grimsby Hospital.

The committee of the Grimsby Hospital, who had decided to place a brass plate and a large photograph in the ward where Jack Travers Cornwell, the young naval hero, died, has received a letter from Mr. F. W. Elwell, a well-known Yorkshire artist, containing the offer of a life-sized portrait of Cornwell in oils, as being a more permanent and more worthy memorial of the brave lad. The offer has been very gratefully accepted by the committee.

### Books for British Prisoners Abroad.

Should British prisoners of war be desirous of carrying on serious reading they can obtain, free of charge, educational books on almost any subject by writing to Mr. A. T. Davies at the Board of Education, Whitehall, London, S.W. Applications should be endorsed by a responsible British officer or N.C.O. in the camp.

### Library as a Hospital.

Arrangements have been made with the Borough Council of Islington for the use of the North Branch Library as a hospital for wounded soldiers. The building practically adjoins the Great Northern Central Hospital, and is now in course of adaptation as an additional military section of eighty beds. When this is completed 365 beds will be available for civilian and military cases in connection with this hospital. The hospital authorities appeal for suitable gifts for the equipment of the Library building.

### Moving a Hospital.

The Church Army Hospital in France is being removed to Scotland to serve as a naval auxiliary hospital.

### Grants in Lieu of Holidays.

As compensation for loss of holidays the employees of the Metropolitan Asylums Board are to receive monetary grants.

### Hospitals and Increased Prices.

At the quarterly meeting of the Governors of the Birmingham Free Hospital for Children, statistics were produced showing that for the first six months of the year the cost of household maintenance was 40 per cent. above the cost before the outbreak of the war.

### Suspension of a Medical Journal.

Our contemporary the *Medical Chronicle*, of Manchester and London, has been temporarily suspended. The journal started in 1884 with some original articles, and under several editorial changes has been specially known for its abstracts from foreign medical journals.

### Games for Wounded Soldiers.

Few things are more appreciated by sick and wounded convalescents who are in hospital than games of all kinds. Realising this, the Wounded Allies Relief Committee, of Sardinia House, Kingsway, W.C., have despatched to French and Belgian military hospitals in France eighteen cases containing such games for the amusement of the patients.

### Button Day in New South Wales.

It is announced that the committee of the War Chest Fund, New South Wales, have been making a special effort to obtain funds through "Button Day," which was fixed for June 30, the aim being to sell to every man and woman a badge or a button at prices varying from 1s. to £1,000. It is stated that several citizens gave £500 each for a button.

### The Late Dr. Austin.

It having been established that the untimely death of Dr. Austin, the medical superintendent at Withington Hospital, was directly due to overwork among the wounded soldiers accommodated there, the Manchester Board of Guardians, who are responsible for the hospital, have decided, subject to the Local Government Board's sanction, to give a sum of £700 to Dr. Austin's widow and children.

### Hospital Friday in New South Wales.

The usual Hospital Saturday Fund changed its historic title and was held in Sydney and elsewhere in New South Wales this year on Friday, May 12. A very large number of ladies in "attractive attire" lent their services, and by pressing their attentions on all passers-by succeeded in amassing over £7,500 during the day. What was the motive of the change, which more resembled a Flag Day than an organised movement?

### "A Dental Engine."

Interesting details regarding a new scheme of trench dentistry were given at the Birmingham Dental Hospital at a meeting of the Central Counties Branch of the British Dental Association. A dental engine, it was stated, had been supplied by the Birmingham Equipment Committee, and, under the superintendence of Captain Bowater, soldiers were being treated in the first-line trenches without having to be sent back to the base. Trench dentistry was reported to be an entire success.

Rates of Subscription: Twelve months, 6/6; Six months, 4/-; Three months, 2/-, post free to any address in the United Kingdom. Abroad, Twelve months, 8/8; Six months, 5/-; Three months, 2/6, post free. The Hospital "Index" to Volume LIX., October 1915 to March 1916, is now ready, price 1½d. per copy, post free. Address The Manager, "The Hospital," The Hospital Building, 28/29 Southampton Street, Strand, London, W.C.

